# P-457. High rates of multidrug resistance in a case series of infants with invasive bacterial infections in Rwanda

**DOI:** 10.1093/ofid/ofaf695.672

**Published:** 2026-01-11

**Authors:** Nanda Ramchandar, Brandon Hadfield, Jean Pierre

**Affiliations:** Naval Medical Center San Diego, San Diego, CA; Rwanda Military Referral and Teaching Hospital, Kigali, Kigali, Rwanda; Rwanda Military Referral and Teaching Hospital, Kigali, Kigali, Rwanda

## Abstract

**Background:**

Neonatal sepsis is a leading cause of mortality in neonates globally. Each year, an estimated 1 million neonates die from bacterial infections, 99% of which occur in low- or middle-income countries (LMIC). Rising global antimicrobial resistance (AMR), has further complicated the management of neonatal sepsis by limiting options for available effective antimicrobial agents. In Rwanda, while there is increasing awareness of rising AMR, there are limited studies describing local pathogens and resistance patterns in neonatal sepsis. This case series provides insight into the etiologic pathogens and associated resistance profiles for neonates admitted at Rwanda Military Referral and Teaching Hospital (RMRTH).

**Table 1.**
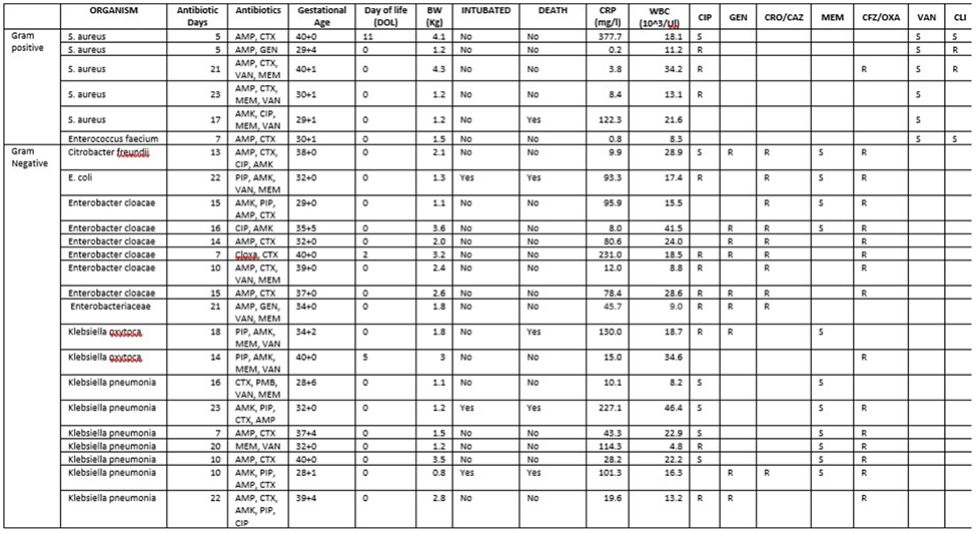
Pathogens recovered from 26 Rwandan infants with an invasive bacterial infection

**Methods:**

We performed a retrospective chart review of all culture positive diagnosed neonatal sepsis at RMRTH from Jan 1, 2022 through 12/31, 2022. We excluded neonates with incomplete medical records and cases where only commensal pathogens were isolated or had cultures without available susceptibility testing. Commensal organisms included all coagulase negative staphylococci and diphtheroids. Demographic and clinical data were extracted from paper charts and the electronic medical records.

**Results:**

Out of 116 neonates with positive cultures, 26 met our inclusion criteria. Among the 26 neonates, 57% (16/26) were preterm (< 37 weeks). Only 11.5% (2/26) needed mechanical ventilation. Most of the neonates, 88.5% (24/26), had cultures done at birth. Microbiological profiles revealed that 69.2% (18/26) of isolated bacteria were gram negative (GN), and that 30.8% (8/26) were gram positive (GP). There were no fungal infections isolated. Among the identified bacteria, the most prevalent species was *Klebsiella pneumonia* (26.9%, 7/26). All isolates of *Klebsiella pneumonia* and *Enterobacter cloacae* species were resistant to gentamicin, cefotaxime, and ceftazidime. 12 of 18 isolates for which susceptibility was available demonstrated ciprofloxacin resistance. The five isolates of *S. aureus* were all methicillin resistant.

**Conclusion:**

In this case series of 26 infants with culture positive sepsis born at a tertiary facility in Rwanda, high rates of antimicrobial resistance were observed. None of the GN isolates recovered were susceptible to a 3^rd^ generation cephalosporin.

**Disclosures:**

All Authors: No reported disclosures

